# miR-152-3p impedes the malignant phenotypes of hepatocellular carcinoma by repressing roundabout guidance receptor 1

**DOI:** 10.1186/s11658-022-00322-y

**Published:** 2022-03-02

**Authors:** Tao Yin, Haonan Zhao

**Affiliations:** Department of General Surgery, Affiliated Hospital of Chifeng University, No. 42 Wangfu Street, Songshan, Chifeng, 024005 China

**Keywords:** Roundabout guidance receptor 1, Prognosis, miR-152-3p, Immunotherapy, Hepatic tumorigenesis

## Abstract

**Background:**

miR-152-3p functions as a tumour suppressor in the progression of hepatic tumorigenesis. Herein, we further discussed the prognostic significance and immune infiltration of miR-152-3p and its potential gene target in hepatocellular carcinoma (HCC).

**Methods:**

The Cancer Genome Atlas (TCGA), Integrative Molecular Database of Hepatocellular Carcinoma (HCCDB), Human Protein Atlas (HPA) and Kaplan–Meier Plotter databases were used to evaluate miR-152-3p and roundabout guidance receptor 1 (*ROBO1*) expression, prognosis and immune infiltration. In vitro cell experiments, including cell proliferation and apoptosis, were evaluated using Cell Counting Kit 8 (CCK8) and terminal-deoxynucleotidyl transferase-mediated nick end labelling (TUNEL) assays.

**Results:**

Up-regulation of *ROBO1* functioned as an oncogene associated with poor prognosis, immune cell enrichment and cell proliferation in HCC. *ROBO1* was significantly positively correlated with the enrichment of multiple immune cells and their biomarkers. Enrichment of type-2 T-helper (Th2) cells is an unfavourable biomarker of HCC prognosis. GSEA revealed that *ROBO1* correlated with apoptosis, mitosis and carcinogenic signalling pathways. Suppression of cell proliferation and the enhancement of cell apoptosis by miR-152-3p mimics were counteracted by overexpression of *ROBO1* in HCC cells.

**Conclusion:**

*ROBO1* expression is positively correlated with multiple immune checkpoint molecules, suggesting that *ROBO1* may be a potential drug target to enhance the potency of immunotherapy. The miR-152-3p/*ROBO1* signalling axis contributes to malignant progression and provides a prospective immunotherapeutic target for HCC.

## Introduction

The biological functions of miR-152-3p are contradictory to the current understanding of the carcinogenesis of multiple malignant tumours [1–3]. miR-152-3p is recognized as an oncosuppressor in breast cancer [1], prostate cancer [3], colorectal cancer [4, 5] and glioma [6, 7]. Conversely, up-regulation of miR-152-3p is revealed in plasma from prostate cancer patients compared with healthy control subjects [2]. In our previous study [8], miR-152-3p repressed cyclin-dependent kinase 8 to restrain hepatic carcinogenesis. Herein, we further explored the roles of miR-152-3p and its gene target in the prognosis and immune infiltration of HCC.

*ROBO1* is a member of the roundabout transmembrane protein receptor family and contributes to axonal guidance in neurogenesis [9, 10]. Recently, ROBO proteins have been implicated in tumour angiogenesis, endothelial cell migration and immune cell recruitment, interacting with Slit2 as a corresponding ligand [11, 12]. The SLIT/ROBO signalling pathway exhibits Janus-faced properties in cancer progression [9]. In several studies, the Slit2/*ROBO1* axis restrains the malignant phenotypes, such as migration, invasion and epithelial–mesenchymal transition, of cancer cells [13–15]. However, up-regulation of *ROBO1* is correlated with poor prognosis and accelerates osteosarcoma cell growth [16]. Moreover, *ROBO1* expression is elevated in nasopharyngeal cancer and is associated with worse overall survival [17]. In HCC, *ROBO1* is up-regulated in tumour tissues and is one of the poor-prognosis-related and immune-related genes that may contribute to hepatic carcinogenesis [18, 19]. *ROBO1* is also substantiated as a serologic marker for the diagnosis of HCC [19].

In our study, miR-152-3p was up-regulated in nine cancer types and down-regulated in five cancer types in a pan-cancer analysis of the TCGA database. Compared with non-tumour tissues, the elevation of *ROBO1* and the reduction of miR-152-3p were observed in HCC tissues. Bioinformatics prediction and experimental measurements validated that *ROBO1* is a direct gene target of miR-152-3p that can repress the protein expression of *ROBO1* in HCC cells. We further investigated the antineoplastic activity, prognosis and immune infiltration of the miR-152-3p/*ROBO1* axis in HCC.

## Materials and methods

### Prediction of miR-152-3p-related gene targets

Three miRNA prediction databases, TargetScan, miRDB and RNA22, were used to predict miR-152-3p-related gene targets. A Venn diagram was utilized to visualize the potential gene targets with R software and the ggplot2 package (version 3.3.3).

### TCGA data analysis

The expression profiles of miR-152-3p and *ROBO1* in pan-cancer or HCC were evaluated using the TCGA database with the ggplot2 package (version 3.3.3). The prognostic analysis was carried out using the TCGA database with the survminer package (version 0.4.9) and the survival package (version 3.2–10) as described previously [20]. In addition, the HCCDB database and the HPA database were used to evaluate *ROBO1* gene expression and immunostaining in HCC tissues, respectively.

### Prognostic analysis of *ROBO1*

The Kaplan–Meier Plotter database (https://kmplot.com/analysis/index.php?p=background) was used to analyse the prognostic significance of *ROBO1* in HCC. In addition, to investigate whether *ROBO1* is an unfavourable factor for OS by mediating the enrichment of immune cells, the Kaplan–Meier Plotter database was used to evaluate OS in the subgroups with enriched and decreased immune cells.

### Immune infiltration

The association between the enrichment of immune cells and *ROBO1* expression in HCC was analysed using the GSVA package (version 1.34.0) with the ssGSEA algorithm [21, 22]. In addition, the correlation of *ROBO1* with immune checkpoint molecules [cytotoxic T-lymphocyte associated protein 4 (*CTLA-4*), programmed cell death 1 (*PDCD-1*), *CD274*, T-cell immunoreceptor with Ig and ITIM domains (*TIGIT*), butyrophilin subfamily 2 member A1 (*BTN2A1*) and *BTN2A2*] was assessed in the TCGA database using the ggplot2 package (version 3.3.3).

### GSEA, GO and KEGG pathway enrichment

An analysis of single-gene differences of *ROBO1* in the TCGA database was prepared for GSEA using the DESeq2 package (version 1.26.0) as described previously [23]. In addition, GO and KEGG pathway enrichment analyses were predicted by the DAVID online database (https://david.ncifcrf.gov/).

### Cell experiments

Cell experiments, including cell culture, cell transfection, luciferase reporter assays (Promega, USA), western blotting (anti-*ROBO1*: ab7279; dilution: 1:500; Abcam) and CCK8 assays (Beyotime), were carried out as described previously [8]. miR-Com-, miR-152-3p-, sh-Con-, sh-*ROBO1*- and *ROBO1*-overexpressing plasmids were obtained from GenePharma (Shanghai, China). A TUNEL kit (Roche) was utilized to analyse cell apoptosis.

### Statistical analysis

The data were analysed using Mann–Whitney *U* test, Wilcoxon signed rank test, one-way analysis of variance log-rank test, univariate Cox regression analysis and Spearman’s correlation analysis.

## Results

### Prediction of gene targets of miR-152-3p

**Fig. 1:**
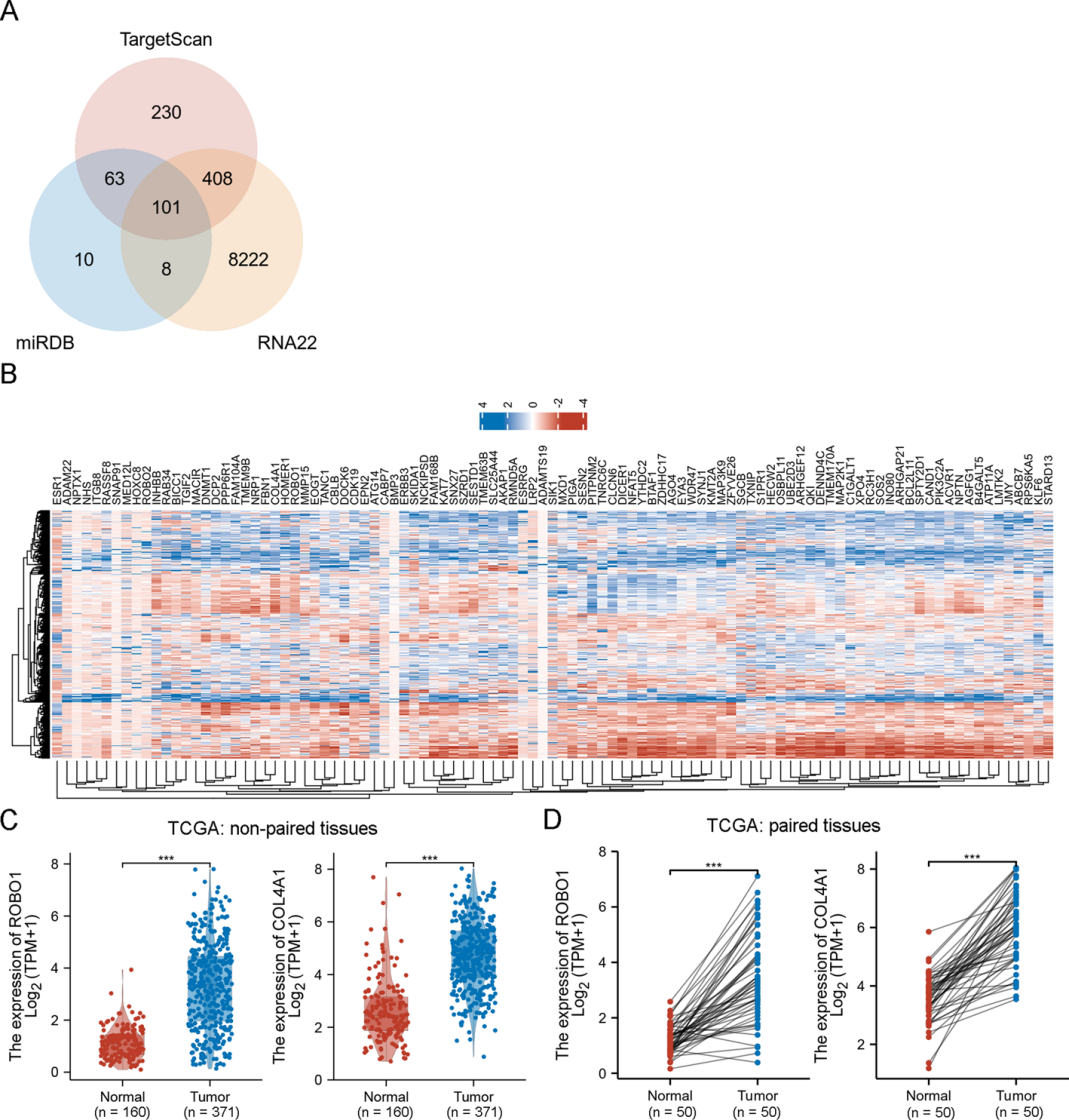
Prediction of gene targets of miR-152-3p. TargetScan, miRDB and RNA22 were implemented to predict gene targets of miR-152-3p (**A**). Heatmap represents the differential expression of 101 gene targets (**B**). *ROBO1* and *COL4A1* expression in non-paired HCC tissues and non-tumour tissues (**C**). *ROBO1* and *COL4A1* expression in paired HCC tissues and adjacent tissues (**D**). ^***^p < 0.001

Based on three miRNA prediction databases, TargetScan, miRDB and RNA22, a total of 101 gene targets of miR-152-3p were collectively identified in those three databases (Fig. 1A). The heatmap shown in Fig. 1B presents the differential expression profiles of the 101 gene targets in the TCGA database, which contained 160 non-tumour tissues and 371 HCC specimens. According to log_2_(fold change) > 2 and *p* < 0.05, *ROBO1* [log_2_(fold change) = 2.21; *p* < 0.001] and *COL4A1* [log_2_(fold change) = 2.07; *p* < 0.001] expression levels were elevated in HCC tissues and filtered out for further investigations (Fig. 1C). In 50 paired tissues, both *ROBO1* and *COL4A1* were expressed at significantly higher levels in cancerous tissues than in paracarcinoma tissues (Fig. 1D).

### Prognostic significance of *ROBO1* and *COL4A1* in HCC

**Fig. 2:**
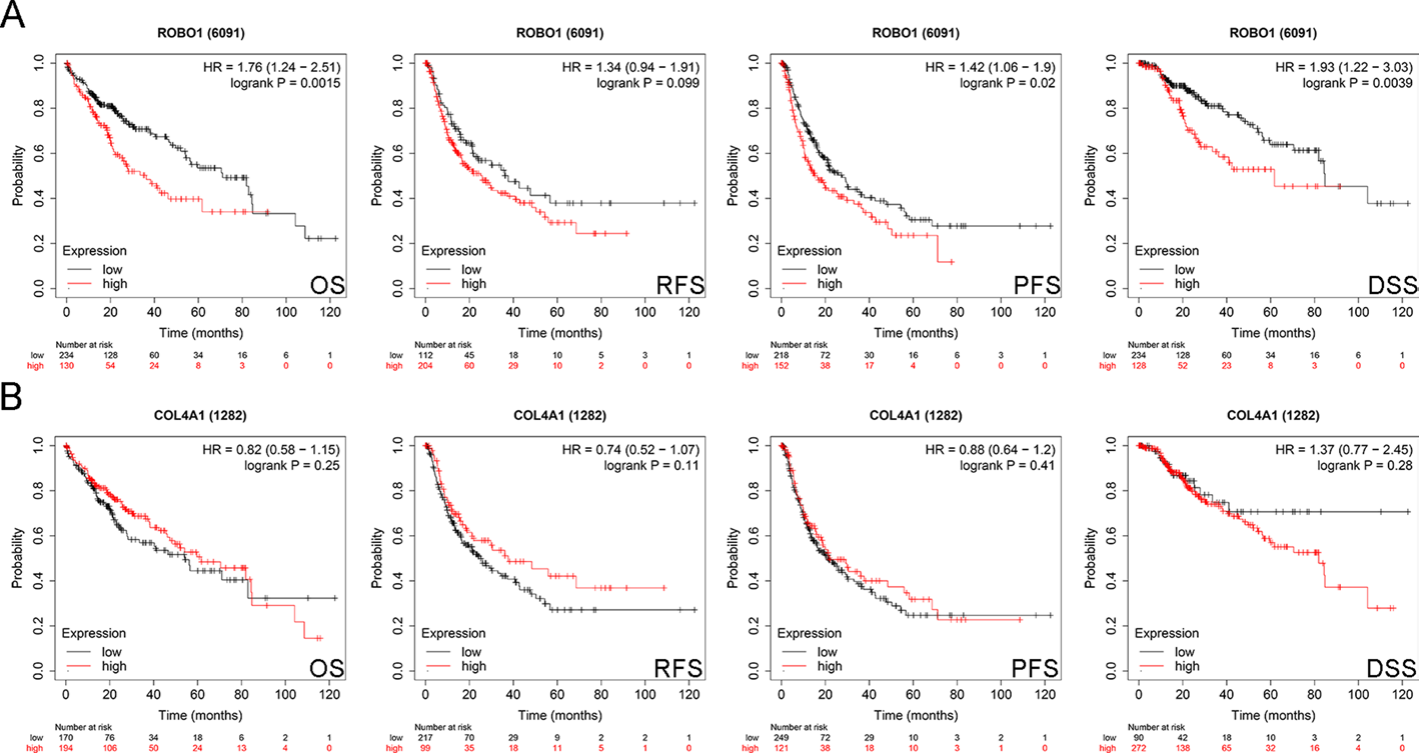
Prognostic significance of *ROBO1* and *COL4A1* in HCC. Kaplan–Meier Plotter database was used to evaluate the prognostic significance of *ROBO1* (**A**) and *COL4A1* (**B**) in HCC

The Kaplan–Meier Plotter database was used to evaluate the association of *ROBO1* and *COL4A1* with prognosis in HCC. Worse OS, PFS and DSS were observed in patients with high *ROBO1* expression than in those with low *ROBO1* expression. However, *COL4A1* expression had no significant correlation with OS, RFS, PFS or DSS in HCC patients (Fig. 2B).

### Further validation of *ROBO1* expression in HCC

**Fig. 3:**
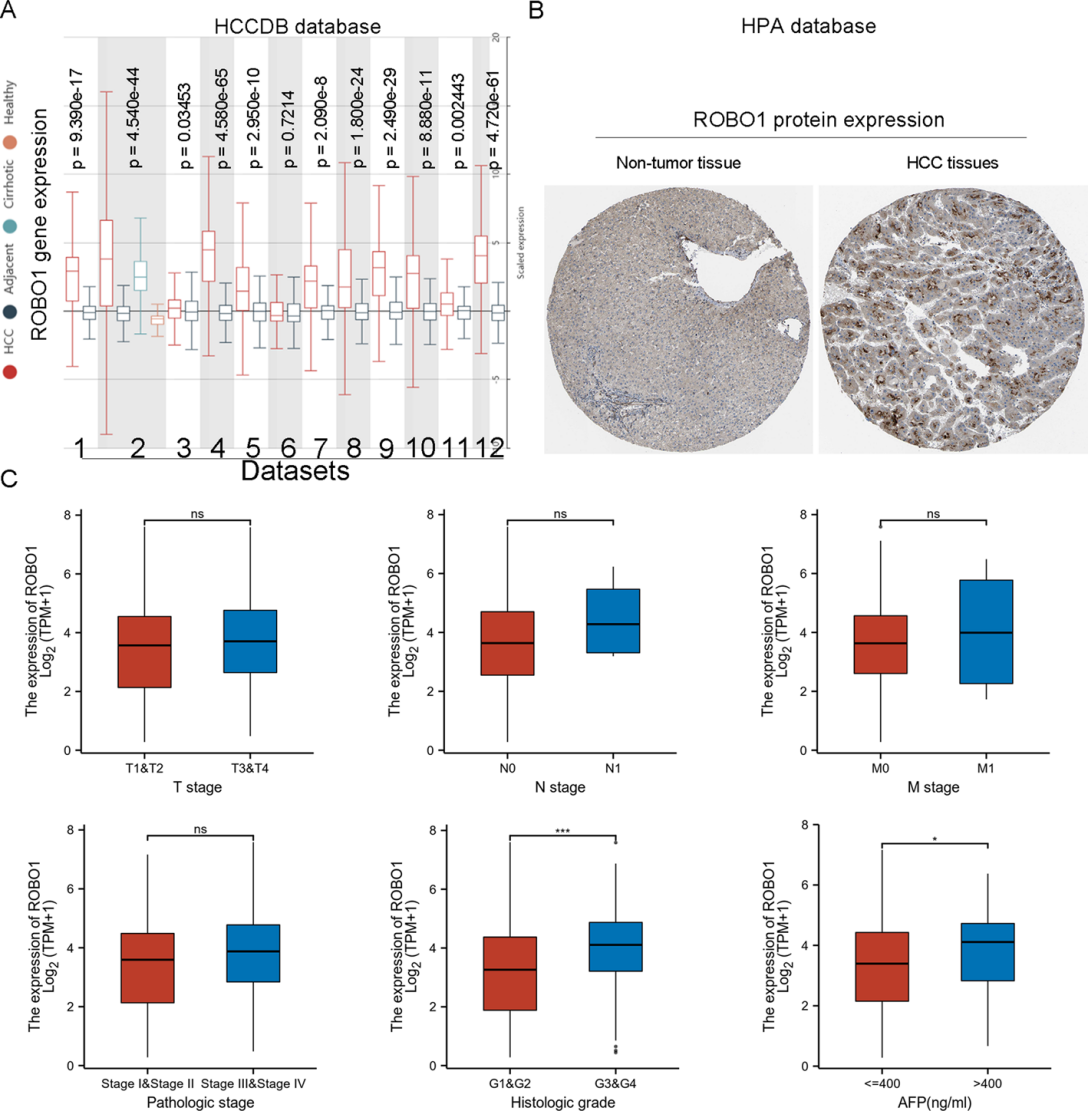
Further validation of *ROBO1* expression in HCC. HCCDB database was utilized to analyse *ROBO1* gene expression (**A**). HPA database revealed prominent positive staining of *ROBO1* (**B**). The association between *ROBO1* expression and clinical parameters in HCC (**C**)

The HCCDB database was utilized to analyse *ROBO1* gene expression in 12 datasets. In 11 of the 12 datasets, up-regulation of *ROBO1* gene expression in HCC tissues was validated by the HCCDB database (Fig. 3A). Intriguingly, the HPA database revealed prominent positive staining of *ROBO1* expression in HCC tissues (Fig. 3B). As shown in Fig. 3C and Table 1, *ROBO1* expression was not significantly different in the T, N, M and pathologic stage subgroups. However, up-regulation of *ROBO1* showed significant correlations with high histologic grade and AFP levels in HCC patients.

**Table 1:** The association of ROBO1 expression with clinical parameters in HCC patients

Characteristic	Low expression of ROBO1	High expression of ROBO1	*p*
*n*	187	187	
Age, *n* (%)			0.797
≤ 60	87 (23.3%)	90 (24.1%)	
> 60	100 (26.8%)	96 (25.7%)	
Gender, *n* (%)			0.507
Female	64 (17.1%)	57 (15.2%)	
Male	123 (32.9%)	130 (34.8%)	
Race, *n* (%)			0.617
Asian	76 (21%)	84 (23.2%)	
Black or African American	10 (2.8%)	7 (1.9%)	
White	94 (26%)	91 (25.1%)	
Pathologic stage, *n* (%)			0.384
Stage I	91 (26%)	82 (23.4%)	
Stage II	40 (11.4%)	47 (13.4%)	
Stage III	36 (10.3%)	49 (14%)	
Stage IV	3 (0.9%)	2 (0.6%)	
T stage, *n* (%)			0.563
T1	97 (26.1%)	86 (23.2%)	
T2	44 (11.9%)	51 (13.7%)	
T3	38 (10.2%)	42 (11.3%)	
T4	5 (1.3%)	8 (2.2%)	
N stage, *n* (%)			1.000
N0	124 (48.1%)	130 (50.4%)	
N1	2 (0.8%)	2 (0.8%)	
M stage, *n* (%)			1.000
M0	132 (48.5%)	136 (50%)	
M1	2 (0.7%)	2 (0.7%)	
Histologic grade, *n* (%)			< 0.001
G1	39 (10.6%)	16 (4.3%)	
G2	95 (25.7%)	83 (22.5%)	
G3	45 (12.2%)	79 (21.4%)	
G4	4 (1.1%)	8 (2.2%)	
AFP (ng/ml), *n* (%)			0.012
≤ 400	123 (43.9%)	92 (32.9%)	
> 400	25 (8.9%)	40 (14.3%)	
Age, median (IQR)	61 (52, 69)	61 (51.25, 68)	0.486

### Relationship of *ROBO1* expression with immune infiltration and immune checkpoint molecules

**Fig. 4:**
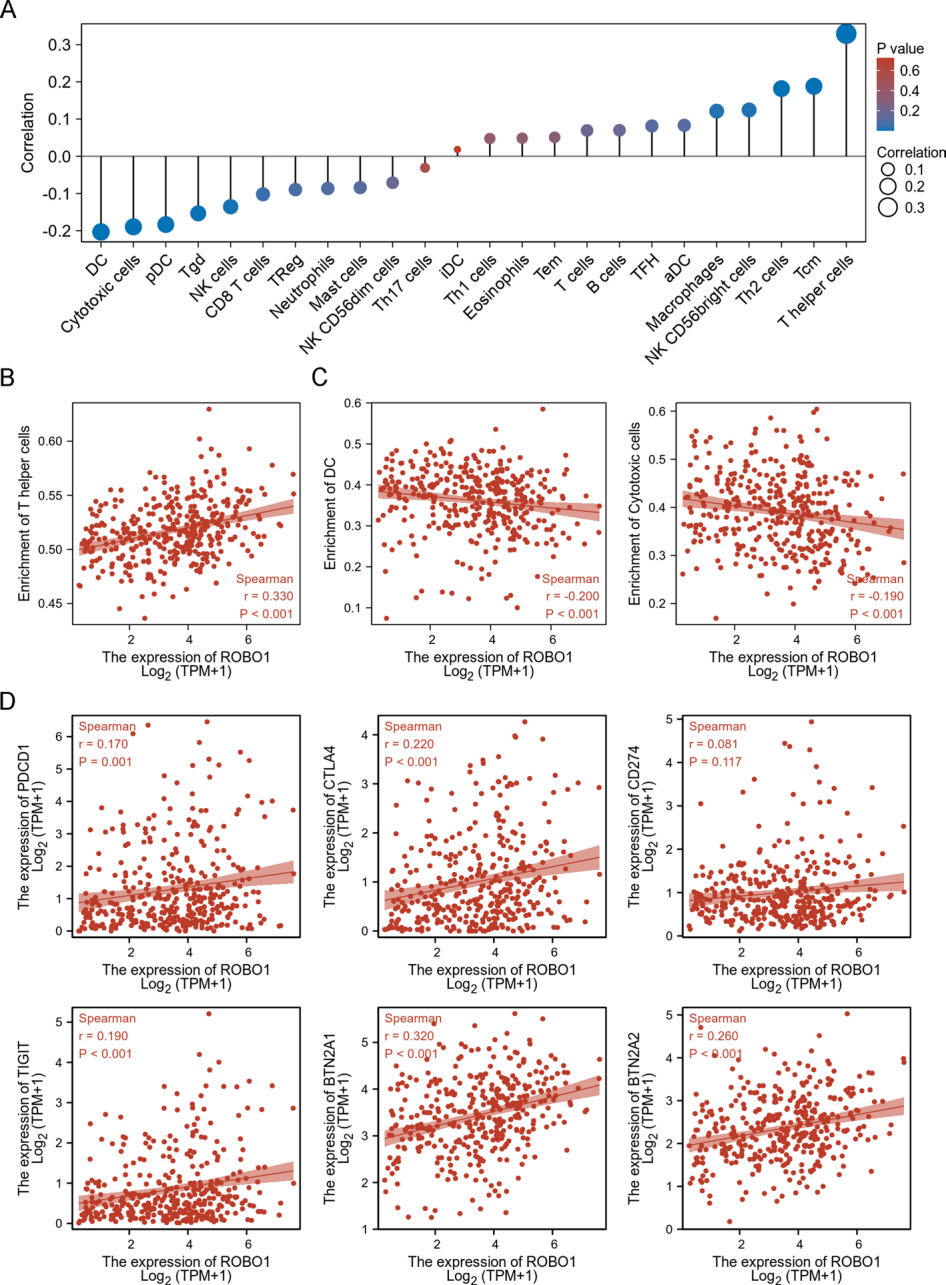
The relationship of *ROBO1* expression with tumour immunology. ssGSEA algorithm was implemented to explore the correlation between *ROBO1* expression and immune cell enrichment in HCC tissues (**A**). Spearman correlation analysis evaluated the correlation of *ROBO1* with T helper cells (**B**), DC and cytotoxic cells (**C)**. Spearman correlation analysis evaluated the correlation of *ROBO1* with 6 immune checkpoint molecules (**D**)

To evaluate whether *ROBO1* expression was associated with tumour immunity, the ssGSEA algorithm was implemented to investigate whether *ROBO1* expression mediates immune cell enrichment in HCC tissues. Spearman correlation analysis revealed that *ROBO1* was positively correlated with five immune cell enrichments and negatively correlated with six immune cell enrichments in HCC tissues (Fig. 4A). The top three correlated immune cells, including T-helper cells (*r* = 0.329; *p* < 0.001), DCs (*r* = −0.203; *p* < 0.001) and cytotoxic cells (*r* = −0.190; *p* < 0.001), are listed in Fig. 4B and C. In addition, *ROBO1* was positively correlated with five immune checkpoint molecules (Fig. 4D), including *PDCD1* (*r* = 0.170; *p* = 0.001), *CTLA4* (*r* = 0.220; *p* < 0.001), *TIGIT* (*r* = 0.190; *p* < 0.001), *BTN2A1* (*r* = 0.320; *p* < 0.001) and *BTN2A2* (*r* = 0.260; *p* < 0.001).

### Prognostic significance of *ROBO1* expression based on immune cell enrichment in HCC patients

**Fig. 5:**
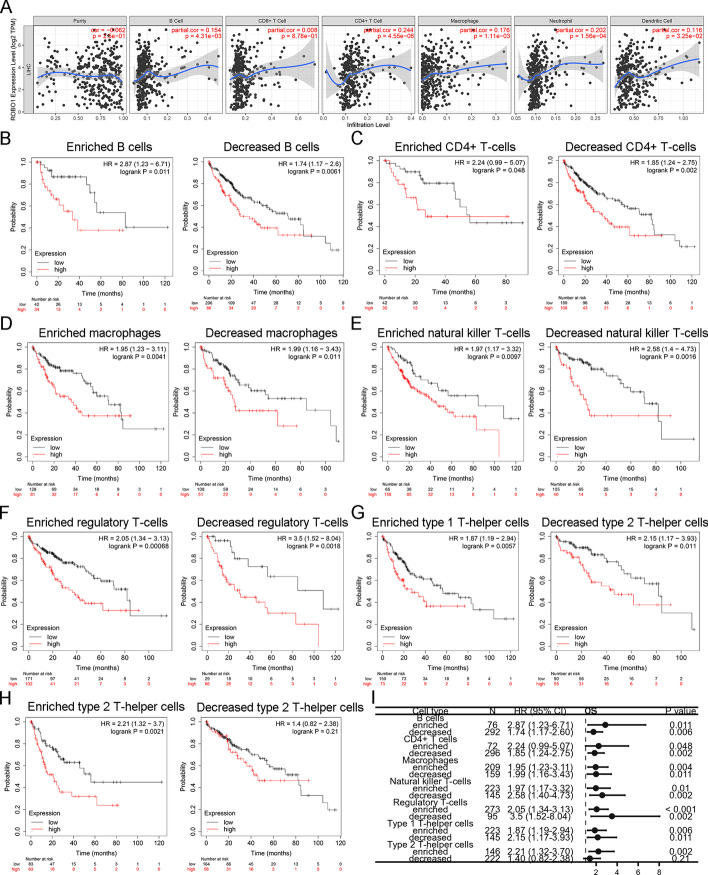
Prognostic significance of *ROBO1* expression based on immune cells enrichment in HCC patients. TIMER database was used to validate the association between *ROBO1* expression and immune cell enrichment in HCC (**A**). The association between *ROBO1* and OS was analysed in subgroups of enriched and decreased immune cells of B cells (**B**), CD4^+^ T cells (**C**), macrophages (**D**), natural killer T cells (**E**), regulatory T cells (**F**) and type-1 T-helper cells (**G**) and type-2 T-helper cells (**H**). Univariate Cox regression was used to analyse the association between *ROBO1* and OS in enriched and decreased immune cell subgroups (**I**)

The TIMER database was also used to validate the association between *ROBO1* expression and immune cell enrichment in HCC. As shown in Fig. 5A, *ROBO1* expression was positively correlated with B cells (*r* = 0.154; *p* = 4.31 × 10^−3^), CD4^+^ T cells (*r* = 0.244; *p* = 4.55 × 10^−6^), macrophages (*r* = 0.176; *p* = 1.11 × 10^−3^), neutrophils (*r* = 0.202; *p* = 1.56 × 10^−4^) and dendritic cells (*r* = 0.116; *p* = 3.25 × 10^−2^). Based on *ROBO1*, in the above-mentioned results, high *ROBO1* expression was correlated with poor prognosis and immune cell enrichment. Therefore, we hypothesized that the *ROBO1*-regulated poor prognosis was associated with immune cell enrichment. The association between *ROBO1* and OS was analysed in the subgroups with enriched and decreased immune cells. In both the enriched and decreased subgroups of B cells (Fig. 5B, I), CD4^+^ T cells (Fig. 5C, I) macrophages (Fig. 5D, I), natural killer T cells (Fig. 5E, I), regulatory T cells (Fig. 5F, I) and type-1 T-helper cells (Fig. 5G, I), high *ROBO1* expression was significantly correlated with poor OS in HCC patients. As shown in Fig. 5H and I, high *ROBO1* expression correlated with poor OS in HCC patients with enriched type-2 T-helper cells, suggesting that poor *ROBO1*-related OS may be partially mediated by the enrichment of type-2 T-helper cells. To further validate the association of *ROBO1* with immune cell enrichment in HCC, the correlation between *ROBO1* and multiple biomarkers of immune cells was evaluated using TCGA database. As shown in Table 2, *ROBO1* was significantly positively correlated with T-helper cell biomarkers (*CXCR3*, *CCR4*, *CCR6* and *CCR10*), Tcm biomarkers (*CD62L*, *CCR5*, *CD58* and *TCF7*), Th2 cell biomarkers (*PTGDR2*, *IL5* and *IL10*) and macrophage biomarkers (*PPARG*, *IRF5* and *CD68*) in HCC.

**Table 2:** The association of ROBO1 expression with the biomarkers of immune cells

Immune cell	Biomarker	*r* value	*p* value
T-helper cell	CXCR3	0.167	0.001
	CCR4	0.222	< 0.001
	CCR6	0.327	< 0.001
	CCR10	0.148	0.004
Tcm	CD62L	0.183	< 0.001
	CCR5	0.201	< 0.001
	CD58	0.354	< 0.001
	TCF7	0.305	< 0.001
Th2 cells	PTGDR2	0.228	< 0.001
	IL5	0.162	0.002
	IL10	0.181	< 0.001
Macrophage	PPARG	0.379	< 0.001
	IRF5	0.224	< 0.001
	CD68	0.179	< 0.001

### Differentially expressed genes in HCC based on *ROBO1* expression

**Fig. 6:**
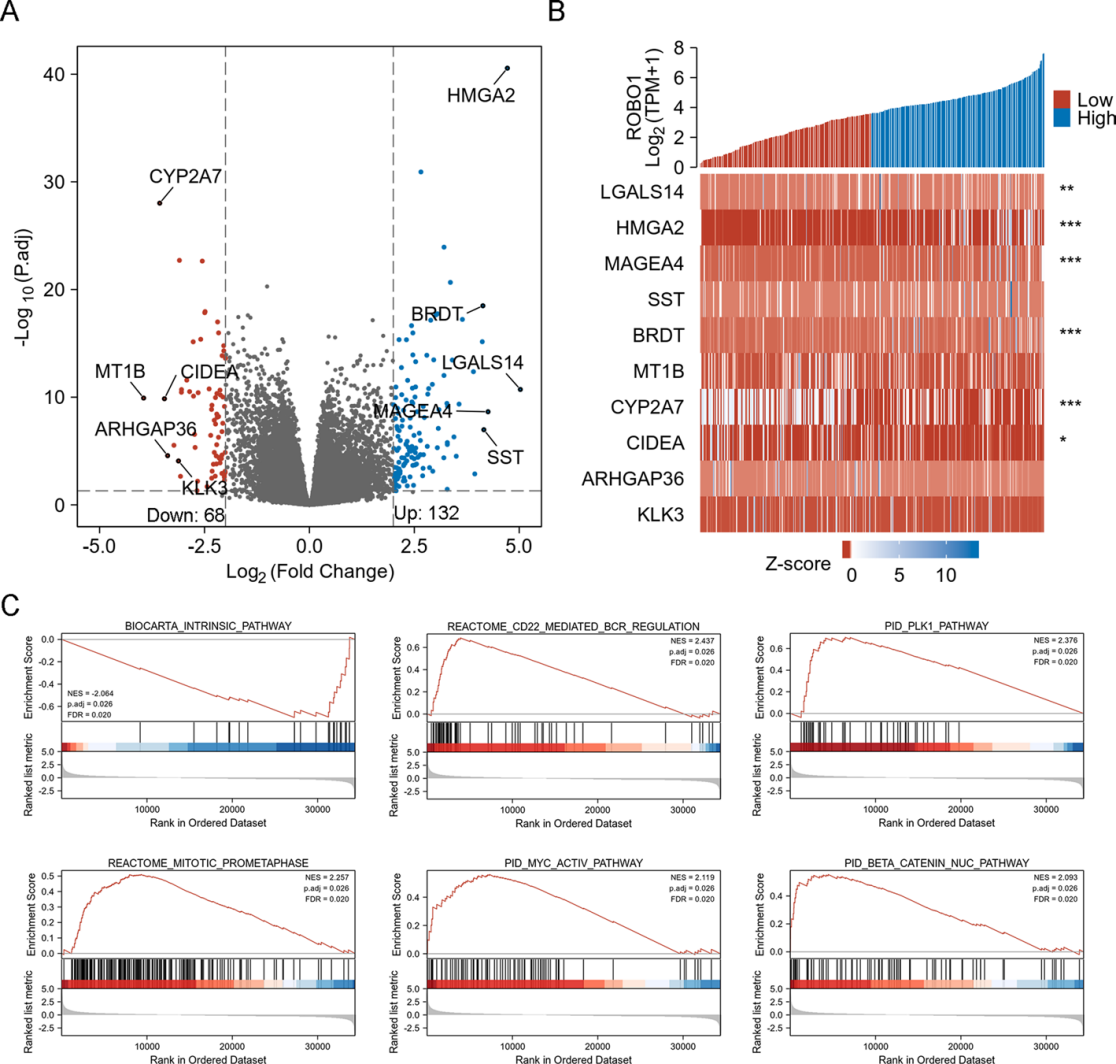
Differentially expressed genes in HCC based on *ROBO1* expression. Differentially expressed genes were categorized on the basis of *ROBO1* high- and low-expression subgroups (**A**). Heatmap presents the expression of the top five up-regulated and down-regulated genes in HCC tissues (**B**). GSEA analysis based on *ROBO1*-related differentially expressed genes in HCC (**C**)

According to |log_2_(fold change)|> 2 and adjusted *p* < 0.05, 132 up-regulated and 68 down-regulated genes were filtered based on the *ROBO1* high- and low-expression subgroups (Fig. 6A). A heatmap was constructed to visualize the expression profiles of the top five up-regulated and down-regulated genes in HCC tissues (Fig. 6B). GSEA suggested that *ROBO1*-related genes were enriched in the Biocarta intrinsic pathway, Reactome CD22 mediator BCR regulation, the PID/PLK1 pathway, Reactome mitotic prometaphase, the PID/MYC activity pathway and the PID/beta-catenin Nuc pathway (Fig. 6C).

### GO and KEGG analyses

**Fig. 7:**
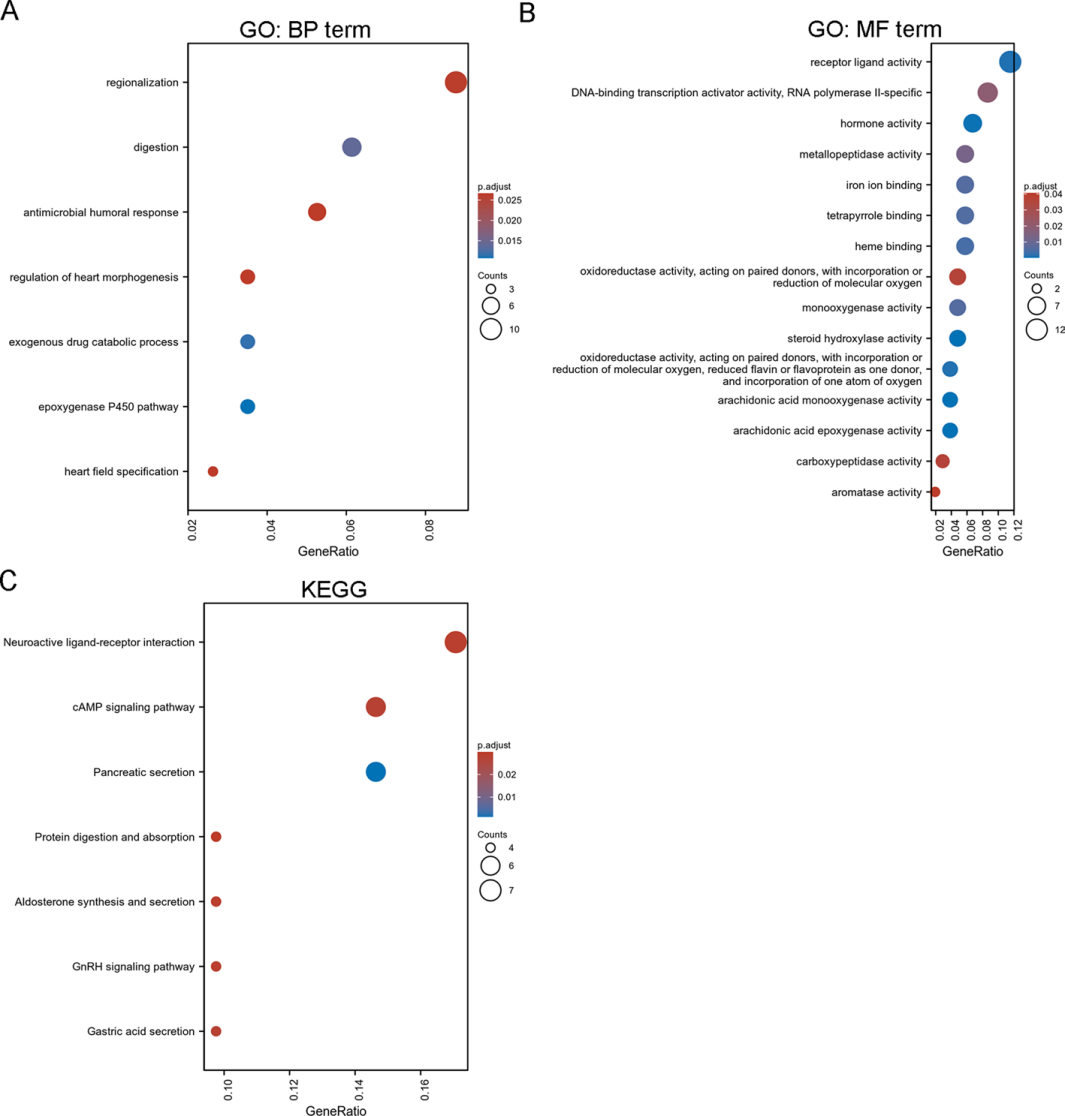
GO and KEGG analysis. *ROBO1*-related top 100 up-regulated and down-regulated genes were used to perform GO [BP terms (**A**) and MF terms (**B**)] and KEGG analysis (**C**)

The *ROBO1*-related top 100 up-regulated and down-regulated genes were used to perform GO and KEGG analyses. Seven BP terms (Fig. 7A), 15 MF terms (Fig. 7B) and 7 KEGG pathways (Fig. 7C) were enriched in HCC.

### Validation of *ROBO1* as a gene target of miR-152-3p

**Fig. 8:**
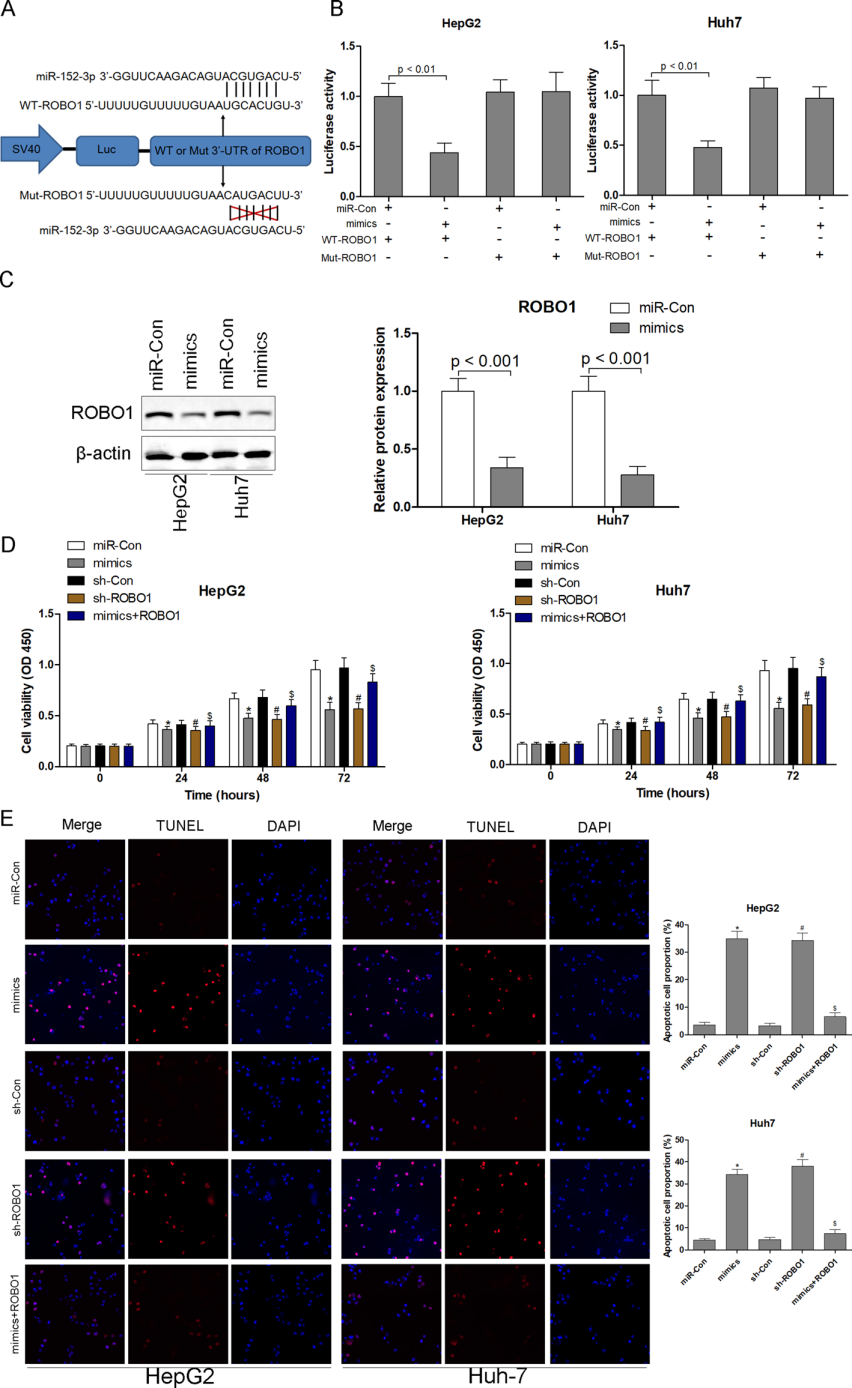
Validation of *ROBO1* as a gene target of miR-152-3p. miR-152-3p binds with the 3′-UTR of *ROBO1* (**A**). In vitro luciferase activity in HepG2 and Huh7 cells (**B**). *ROBO1* protein expression in HepG2 and Huh7 cells with miR-152-3p mimics transfection (**C**). Cell proliferation (**D**) and apoptosis (**E**) of HepG2 and Huh7 cells were analysed using CCK8 and TUNEL. ^*^p < 0.05 versus miR-Con group; ^#^*p* < 0.05 versus sh-Con group; ^$^*p* < 0.05 versus mimics group

The analyses performed in the online databases revealed that *ROBO1* might be a potential gene target of miR-152-3p, which was predicted to bind with the 3′-UTR of *ROBO1* (Fig. 8A). In vitro luciferase activity was significantly diminished in HepG2 and Huh7 cells after transfection with miR-152-3p mimics compared with the control group (Fig. 8B), indicating that miR-152-3p can directly bind with *ROBO1*. Western blot analysis revealed significant decreases in *ROBO1* protein expression in HepG2 and Huh7 cells transfected with miR-152-3p mimics (Fig. 8C). To investigate the roles of miR-152-3p and *ROBO1* in the progression of HCC, miR-152-3p mimics, sh-*ROBO1* or *ROBO1* overexpression plasmids were delivered into HepG2 and Huh7 cells. As shown in Fig. 8D and E, transfection of miR-152-3p mimics or sh-*ROBO1* inhibited cell proliferation and induced cell apoptosis of HepG2 and Huh7 cells. However, the inhibition of cell proliferation and the enhancement of cell apoptosis by miR-152-3p mimics were counteracted by overexpression of *ROBO1* in HepG2 and Huh7 cells (Fig. 8D, E).

### The association between miR-152-3p and HCC

**Fig. 9:**
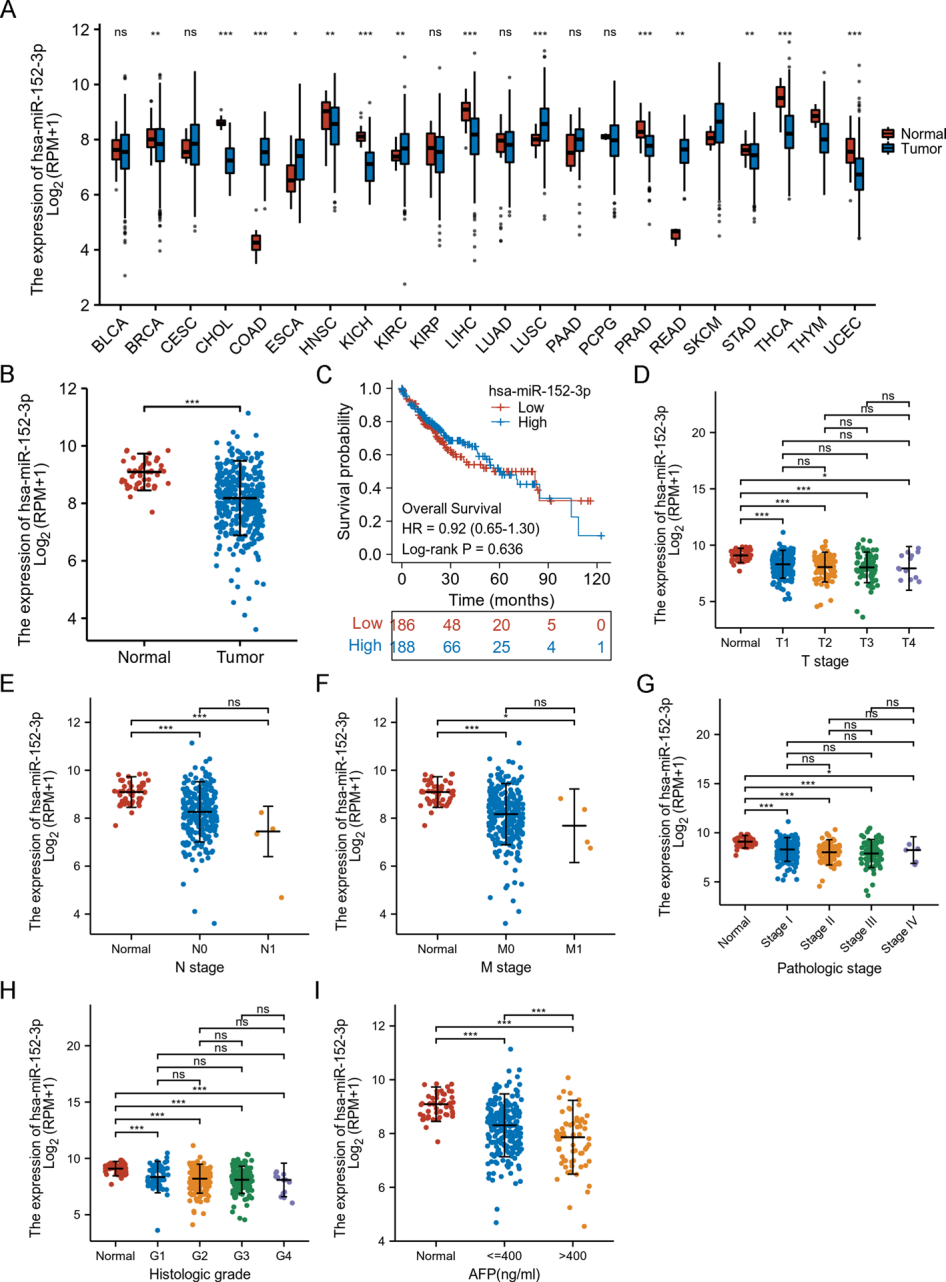
Clinical significance of miR-152-3p in HCC. miR-152-3p expression in pan-cancer analysis of TCGA database (**A**). miR-152-3p expression in TCGA database (**B**). The association between miR-152-3p and OS (**C**), T (**D**), N (**E**), M (**F**) and pathologic stage (**G**), and histologic grade (**H**). miR-152-3p low expression was significantly correlated with high AFP levels in HCC patients (**I**). ^*^p < 0.05; ^***^p < 0.001

As shown in Fig. 9A, miR-152-3p was up-regulated in nine cancer types and down-regulated in five cancer types in pan-cancer analysis of the TCGA database. Compared with non-tumour tissues, miR-152-3p was significantly down-regulated in HCC tissues (Fig. 9B), while miR-152-3p expression had no significant correlation with OS (Fig. 9C), T stage (Fig. 9D), N stage (Fig. 9E), M stage (Fig. 9F), pathologic stage (Fig. 9G) or histologic grade (Fig. 9H). As shown in Fig. 9I, low miR-152-3p expression was significantly correlated with high AFP levels in HCC patients.

### The association between miR-152-3p and immune infiltration

**Fig. 10:**
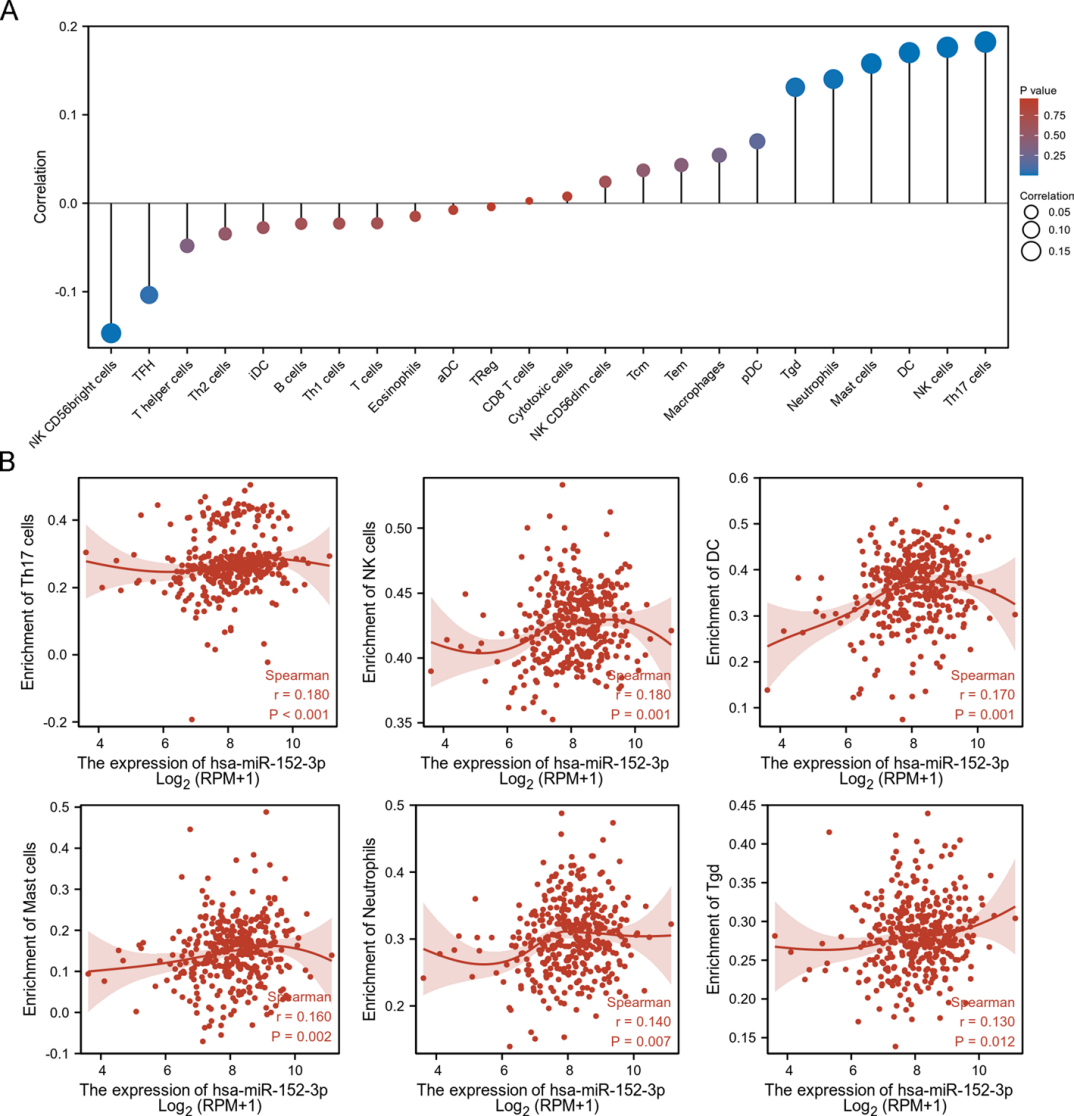
The association between miR-152-3p and immune infiltration. ssGSEA algorithm was implemented to evaluate miR-152-3p mediated immune cell enrichment (**A**). The top six correlated immune cells, including Th17 cells, NK cells, DC, mast cells, neutrophils and Tgd (**B**)

To determine whether miR-152-3p expression was associated with tumour immunity, the ssGSEA algorithm was implemented to explore the correlation between miR-152-3p expression and immune cell enrichment in HCC tissues. Spearman correlation analysis revealed that *ROBO1* was positively correlated with six immune cell enrichments and negatively correlated with two immune cell enrichments in HCC tissues (Fig. 10A). The top six correlated immune cells, including Th17 cells (*r* = 0.180; *p* < 0.001), NK cells (*r* = 0.180; *p* = 0.001), DCs (*r* = 0.170; *p* = 0.001), mast cells (*r* = 0.160; *p* = 0.002), neutrophils (*r* = 0.140; *p* = 0.007) and Tgd (*r* = 0.130; *p* = 0.012), are shown in Fig. 10B.

## Discussion

Our findings revealed a novel signalling axis, miR-152-3p/*ROBO1*, that contributes to hepatic tumorigenesis by regulating cell proliferation and apoptosis in vitro and mediates immune cell enrichment in HCC. *ROBO1* was identified as an oncogene to accelerate the enrichment of type-2 T-helper cells that may be correlated with poor prognosis in patients with HCC.

The convertible role of *ROBO1* as a tumour suppressor or an oncogene has been observed in different cancer types [15, 24–26]. At present, the transcription level and functions of *ROBO1* are consistent in the progression of HCC [19, 27, 28]. For example, the enhancement of *ROBO1* expression triggers tumour growth, invasion and metastasis in HCC Sk-hep-1 cells [27]. Down-regulation of *ROBO1* by miR-490-5p contributes to the induction of apoptosis and inhibits malignant phenotypes in HCC Hep3B cells [28]. Hirotaka et al. substantiated that *ROBO1* is overexpressed in the serum of HCC patients, HCC tissues and cell lines and may be a serologic marker for the diagnosis of HCC [19]. In our study, marked elevations in *ROBO1* gene and protein expression were corroborated by three databases, TCGA, HCCDB and HPA, in HCC tissues, and *ROBO1*, as an unfavourable prognostic marker, was correlated with worse OS, PFS and DSS. We also found that *ROBO1* loss of function augmented the proportion of apoptotic cells and restrained the proliferation of HCC cell lines.

Diversiform immune cells are an important component of the tumour microenvironment and correlate with prognosis, metastasis and immunotherapy in cancer [29, 30]. HCC is associated with inflammatory processes, such as viral infection and cirrhosis, which drive the enrichment of immune cells, especially lymphocytes, contributing to poor prognosis [30, 31]. In our study, both the TCGA and TIMER databases indicated that *ROBO1* was significantly and positively correlated with macrophages in HCC tissues. Tumour-associated macrophages (TAMs) contribute to the initiation and progression of HCC by secreting pro-inflammatory cytokines and triggering the expansion of cancer stem cells [32]. In addition, TAMs facilitate metastasis and predict poor prognosis in patients with HCC [32, 33].

Our findings also suggested that poor OS in *ROBO1*HCC patients with high *ROBO1* expression was associated with the enrichment of Th2 cells. The TCGA database also validated a significant positive correlation (*r* = 0.182; *p* < 0.001) between *ROBO1* and Th2 cells in HCC tissues. Enrichment of Th2 cells has frequently been reported in HCC patients and is implicated in tumour invasion and metastasis [34, 35]. Duan et al. indicated that inhibition of Th2 cell activity by immune checkpoint blockades (ICBs) may be associated with increased survival time and decreased tumour recurrence in a mouse model of HCC [36]. These findings suggested that enrichment of Th2 cells represented an unfavourable biomarker of HCC prognosis.

ICBs are a class of antitumour immunotherapeutic drugs that suppress immune checkpoint molecules, such as PD-1, PD-L1, CTLA-4 and TIGIT, to restore immune recognition and immunogenicity in HCC [37–39]. Our findings suggested that *ROBO1* was positively correlated with five immune checkpoint molecules, *PDCD1*, *CTLA4*, *TIGIT*, *BTN2A1* and *BTN2A*, in HCC, reflecting that *ROBO1* inhibitors may have a synergistic effect and enhance the potency of ICBs to improve therapeutic efficiency in HCC patients.

In this study, both predictions and experiments corroborated that *ROBO1* is a direct gene target of miR-152-3p. In our previous study [8], miR-152-3p expression was decreased in HCC tissues, and overexpression of miR-152-3p targeted cyclin-dependent kinase 8 to mediate antineoplastic activity in HCC. Other studies have also shown that miR-152-3p possesses outstanding anticancer properties in colorectal cancer, prostate cancer and lymphoma [5, 40, 41]. Based on our previous findings [8] and the present results, miR-152-3p may function as a tumour suppressor by mediating multiple gene targets to prevent hepatic tumorigenesis.

In conclusion, *ROBO1* was identified as an unfavourable prognostic marker and was correlated with the enrichment of Th2 cells in HCC. *ROBO1* expression was also positively correlated with multiple immune checkpoint molecules, suggesting that *ROBO1* may be a potential drug target to enhance the potency of immunotherapy. Further, *ROBO1* was identified as a direct target of miR-152-3p, indicating that the miR-152-3p/*ROBO1* signalling axis may be involved in the pathogenesis of hepatic tumorigenesis.

## Data Availability

The data that support the findings of this study are available from the corresponding author upon reasonable request.

## Abbreviations

HCC: Hepatocellular carcinoma
TCGA: The Cancer Genome Atlas
HCCDB: Integrative molecular database of hepatocellular carcinoma
HPA: Human Protein Atlas
*ROBO1*: Roundabout guidance receptor 1
CCK-8: Cell Counting Kit 8
TUNEL: Terminal-deoxynucleotidyl transferase-mediated nick end labelling
Th2: Type 2 T-helper

## References

[1] Zeng K, He B, Yang BB, Xu T, Chen X, Xu M (2018). The pro-metastasis effect of circANKS1B in breast cancer. Mol Cancer.

[2] Moya L, Meijer J, Schubert S, Matin F, Batra J (2019). Assessment of miR-98-5p, miR-152-3p, miR-326 and miR-4289 expression as biomarker for prostate cancer diagnosis. Int J Mol Sci.

[3] Ramalho-Carvalho J, Gonçalves CS, Graça I, Bidarra D, Pereira-Silva E, Salta S (2018). A multiplatform approach identifies miR-152-3p as a common epigenetically regulated onco-suppressor in prostate cancer targeting TMEM97. Clin Epigenet.

[4] Wang C, Ma X, Zhang J, Jia X, Huang M (2020). DNMT1 maintains the methylation of miR-152-3p to regulate TMSB10 expression, thereby affecting the biological characteristics of colorectal cancer cells. IUBMB Life.

[5] Liu X, Li L, Bai J, Li L, Fan J, Fu Z (2021). lncRNA PVT1 promotes progression of colorectal cancer by sponging miR-152-3p and regulating E2F3/MAPK8 signaling. Cancer Sci.

[6] Shi J, Zhang Y, Qin B, Wang Y, Zhu X (2019). Long non-coding RNA LINC00174 promotes glycolysis and tumor progression by regulating miR-152-3p/SLC2A1 axis in glioma. J Exp Clin Cancer Res.

[7] Sun J, Tian X, Zhang J, Huang Y, Lin X, Chen L (2017). Regulation of human glioma cell apoptosis and invasion by miR-152-3p through targeting DNMT1 and regulating NF2: MiR-152-3p regulate glioma cell apoptosis and invasion. J Exp Clin Cancer Res.

[8] Yin T, Liu MM, Jin RT, Kong J, Wang SH, Sun WB (2019). miR-152–3p modulates hepatic carcinogenesis by targeting cyclin-dependent kinase 8. Pathol Res Pract.

[9] Jiang Z, Liang G, Xiao Y, Qin T, Chen X, Wu E (2019). Targeting the SLIT/ROBO pathway in tumor progression: molecular mechanisms and therapeutic perspectives. Ther Adv Med Oncol.

[10] Rama N, Dubrac A, Mathivet T, Chárthaigh RAN, Genet G, Cristofaro B (2015). Slit2 signaling through *ROBO1* and Robo2 is required for retinal neovascularization. Nat Med.

[11] Legg JA, Herbert JM, Clissold P, Bicknell R (2008). Slits and roundabouts in cancer, tumour angiogenesis and endothelial cell migration. Angiogenesis.

[12] Koohini Z, Koohini Z, Teimourian S (2019). Slit/Robo signaling pathway in cancer; a new stand point for cancer treatment. Pathol Oncol Res.

[13] Xia Y, Wang L, Xu Z, Kong R, Wang F, Yin K (2019). Reduced USP33 expression in gastric cancer decreases inhibitory effects of Slit2-*ROBO1* signalling on cell migration and EMT. Cell Prolif.

[14] Xu Y, Li WL, Fu L, Gu F, Ma YJ (2010). Slit2/*ROBO1* signaling in glioma migration and invasion. Neurosci Bull.

[15] Feng Y, Feng L, Yu D, Zou J, Huang Z (2016). srGAP1 mediates the migration inhibition effect of Slit2-*ROBO1* in colorectal cancer. J Exp Clin Cancer Res.

[16] Zhao SJ, Shen YF, Li Q, He YJ, Zhang YK, Hu LP (2018). SLIT2/*ROBO1* axis contributes to the Warburg effect in osteosarcoma through activation of SRC/ERK/c-MYC/PFKFB2 pathway. Cell Death Dis.

[17] Alajez NM, Lenarduzzi M, Ito E, Hui AB, Shi W, Bruce J (2011). MiR-218 suppresses nasopharyngeal cancer progression through downregulation of survivin and the SLIT2-*ROBO1* pathway. Cancer Res.

[18] Wu L, Quan W, Luo Q, Pan Y, Peng D, Zhang G (2020). Identification of an immune-related prognostic predictor in hepatocellular carcinoma. Front Mol Biosci.

[19] Ito H, Funahashi S, Yamauchi N, Shibahara J, Midorikawa Y, Kawai S (2006). Identification of *ROBO1* as a novel hepatocellular carcinoma antigen and a potential therapeutic and diagnostic target. Clin Cancer Res.

[20] Liu J, Lichtenberg T, Hoadley KA, Poisson LM, Lazar AJ, Cherniack AD (2018). An integrated TCGA pan-cancer clinical data resource to drive high-quality survival outcome analytics. Cell.

[21] Hänzelmann S, Castelo R, Guinney J (2013). GSVA: gene set variation analysis for microarray and RNA-seq data. BMC Bioinform.

[22] Bindea G, Mlecnik B, Tosolini M, Kirilovsky A, Waldner M, Obenauf AC (2013). Spatiotemporal dynamics of intratumoral immune cells reveal the immune landscape in human cancer. Immunity.

[23] Love MI, Huber W, Anders S (2014). Moderated estimation of fold change and dispersion for RNA-seq data with DESeq2. Genome Biol.

[24] Parray A, Siddique HR, Kuriger JK, Mishra SK, Rhim JS, Nelson HH (2014). *ROBO1*, a tumor suppressor and critical molecular barrier for localized tumor cells to acquire invasive phenotype: study in African–American and Caucasian prostate cancer models. Int J Cancer.

[25] Le LT, Cazares O, Mouw JK, Chatterjee S, Macias H, Moran A (2016). Loss of miR-203 regulates cell shape and matrix adhesion through *ROBO1*/Rac/FAK in response to stiffness. J Cell Biol.

[26] Tie J, Pan Y, Zhao L, Wu K, Liu J, Sun S (2010). MiR-218 inhibits invasion and metastasis of gastric cancer by targeting the *ROBO1* receptor. PLoS Genet.

[27] Yuan M, Guo H, Li J, Sui C, Qin Y, Wang J (2016). Slit2 and *ROBO1* induce opposing effects on metastasis of hepatocellular carcinoma Sk-hep-1 cells. Int J Oncol.

[28] Chen W, Ye L, Wen D, Chen F (2019). MiR-490-5p inhibits hepatocellular carcinoma cell proliferation, migration and invasion by directly regulating *ROBO1*. Pathol Oncol Res.

[29] Ringelhan M, Pfister D, O'Connor T, Pikarsky E, Heikenwalder M (2018). The immunology of hepatocellular carcinoma. Nat Immunol.

[30] Lu C, Rong D, Zhang B, Zheng W, Wang X, Chen Z (2019). Current perspectives on the immunosuppressive tumor microenvironment in hepatocellular carcinoma: challenges and opportunities. Mol Cancer.

[31] Ally A, Balasundaram M, Carlsen R, Chuah E, Clarke A, Dhalla N (2017). Comprehensive and integrative genomic characterization of hepatocellular carcinoma. Cell.

[32] Wan S, Zhao E, Kryczek I, Vatan L, Sadovskaya A, Ludema G (2014). Tumor-associated macrophages produce interleukin 6 and signal via STAT3 to promote expansion of human hepatocellular carcinoma stem cells. Gastroenterology.

[33] Wu J, Gao W, Tang Q, Yu Y, You W, Wu Z (2021). M2 macrophage-derived exosomes facilitate HCC metastasis by transferring α(M) β(2) integrin to tumor cells. Hepatology.

[34] Zhu HF, Liu YP, Liu DL, Ma YD, Hu ZY, Wang XY (2019). Role of TGFβ3-Smads-Sp1 axis in DcR3-mediated immune escape of hepatocellular carcinoma. Oncogenesis.

[35] Matsui T, Nagai H, Sumino Y, Miki K (2008). Relationship of peripheral blood CD4-positive T cells to carcinogenesis in patients with HCV-related chronic hepatitis and liver cirrhosis. Cancer Chemother Pharmacol.

[36] Duan X, Wang M, Han X, Ren J, Huang G, Ju S (2020). Combined use of microwave ablation and cell immunotherapy induces nonspecific immunity of hepatocellular carcinoma model mice. Cell Cycle.

[37] Xu F, Jin T, Zhu Y, Dai C (2018). Immune checkpoint therapy in liver cancer. J Exp Clin Cancer Res.

[38] Pinter M, Jain RK, Duda DG (2021). The current landscape of immune checkpoint blockade in hepatocellular carcinoma: a review. JAMA Oncol.

[39] Liang L, Ge K, Zhang F, Ge Y (2018). The suppressive effect of co-inhibiting PD-1 and CTLA-4 expression on H22 hepatomas in mice. Cell Mol Biol Lett.

[40] Tao LJ, Pan XY, Wang JW, Zhang L, Tao LS, Liang CZ (2021). Circular RNA circANKS1B acts as a sponge for miR-152-3p and promotes prostate cancer progression by upregulating TGF-α expression. Prostate.

[41] Tian Y, Li L, Lin G, Wang Y, Wang L, Zhao Q (2021). lncRNA SNHG14 promotes oncogenesis and immune evasion in diffuse large-B-cell lymphoma by sequestering miR-152-3p. Leuk Lymphoma.

